# The Next Generation of Influenza Vaccines: Towards a Universal Solution

**DOI:** 10.3390/vaccines9010026

**Published:** 2021-01-07

**Authors:** Christopher L.D. McMillan, Paul R. Young, Daniel Watterson, Keith J. Chappell

**Affiliations:** 1 School of Chemistry and Molecular Biosciences, The University of Queensland, St Lucia, QLD 4072, Australia; p.young@uq.edu.au (P.R.Y.); d.watterson@uq.edu.au (D.W.); 2 The Australian Institute for Biotechnology and Nanotechnology, The University of Queensland, St Lucia, QLD 4072, Australia; 3 The Australian Infectious Disease Research Centre, The University of Queensland, St Lucia, QLD 4072, Australia

**Keywords:** influenza, vaccine, universal influenza vaccine, hemagglutinin, pandemic

## Abstract

Influenza viruses remain a constant burden in humans, causing millions of infections and hundreds of thousands of deaths each year. Current influenza virus vaccine modalities primarily induce antibodies directed towards the highly variable head domain of the hemagglutinin protein on the virus surface. Such antibodies are often strain-specific, meaning limited cross-protection against divergent influenza viruses is induced, resulting in poor vaccine efficacy. To attempt to counteract this, yearly influenza vaccination with updated formulations containing antigens from more recently circulating viruses is required. This is an expensive and time-consuming exercise, and the constant arms race between host immunity and virus evolution presents an ongoing challenge for effective vaccine development. Furthermore, there exists the constant pandemic threat of highly pathogenic avian influenza viruses with high fatality rates (~30–50%) or the emergence of new, pathogenic reassortants. Current vaccines would likely offer little to no protection from such viruses in the event of an epidemic or pandemic. This highlights the urgent need for improved influenza virus vaccines capable of providing long-lasting, robust protection from both seasonal influenza virus infections as well as potential pandemic threats. In this narrative review, we examine the next generation of influenza virus vaccines for human use and the steps being taken to achieve universal protection.

## 1. Introduction

Influenza viruses are *Orthomyxovirus* species belonging to the *Orthomyxoviridae* family [1]. While influenza viruses can be classified into four genera (A, B, C and D), only influenza A and B viruses cause clinical disease in humans [2,3,4,5]. In humans, influenza viruses infect the respiratory tract, with symptoms including high fever, dry cough, headache, malaise, rhinorrhea and myalgia, with death as the outcome in severe cases [6]. Influenza viruses cause up to 650,000 deaths globally each year, with 5–20% of the human population contracting non-lethal infections annually [7].

**Figure 1:**
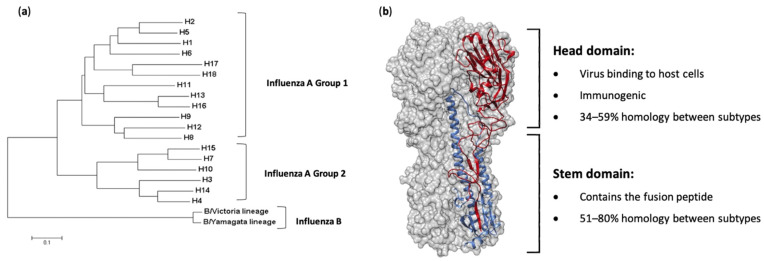
Phylogeny and structure of influenza virus hemagglutinin. (**a**) A rooted phylogenetic tree based on the amino acid sequences of hemagglutinin (HA) sequences from influenza A and B viruses, adapted from Noh et al. [12]. (**b**) The structure of the ectodomain of the influenza hemagglutinin protein from the A/California/04/2009(H1N1) virus (PDB ID 3LZG). One HA monomer is shown with the HA_1_ subunit in red and the HA_2_ subunit in blue.

Influenza viruses are characterized based on their surface proteins—hemagglutinin (HA) and neuraminidase (NA). There are currently 18 known serotypes/antigenic types of HA (Figure 1a) and 11 known of NA. Almost all human influenza infections are caused by H1- and H3-containing strains (H1N1 and H3N2), with two lineages of influenza B virus—Victoria and Yamagata—also circulating globally on a seasonal basis [7]. Of particular concern, however, are zoonotic events in which highly pathogenic avian influenza viruses such as H5N1, H7N9 and H9N2 viruses are transmitted from avian species to humans. Although limited human-to-human transmission is observed, case fatality rates are significantly higher than seasonal infections (e.g., H5N1, ~53%; H7N9, ~32%) [8]. Furthermore, evidence suggests only minor genetic changes are required to allow increased replication rates in human cells, which could lead to more efficient human-to-human transmission and a subsequent pandemic [9,10]. Additionally, emergence of reassortant viruses whereby entire genome segments are reassorted between viruses upon co-infection of a host with different strains is another mechanism by which pandemic influenza viruses can emerge. Such an event was responsible for the H1N1 pandemic of 2009 and has seen H5N6 viruses emerge in aquatic duck populations more recently [11].

To control the impacts of influenza virus infections, vaccination is the best possible intervention. The influenza HA protein (Figure 1b) has been the primary target for vaccines, as it is large, readily accessible on the virus surface, is essential for virus binding and infection of host cells and is the major target of neutralizing antibodies [13]. While antibodies are produced against other influenza proteins during virus infection, and some of these antigens have been trialed previously as vaccine candidates, anti-HA antibodies are the most abundant and protective [14,15,16,17,18]. As such, current licensed influenza vaccines aim to induce HA-specific antibodies. Traditional influenza vaccines, of which approximately 500–800 million doses are produced annually [19], are created by inactivation and splitting of influenza viruses that have been propagated in hens’ embryonated eggs. Each vaccine dose is measured by HA content, and a standard adult dose contains from 15 to 60 µg of each HA (i.e., H1, H3 and either one or the two HAs from the two influenza B lineages, depending on whether it is a tri- or quadrivalent vaccine) [20]. In people over 65 years of age, high-dose vaccines containing 60 µg of HA from each strain, or suitably adjuvanted influenza vaccines, are recommended [21]. The virion surface-displayed HA molecule is a virion membrane-anchored trimer that comprises two structural elements: a distal, highly variable head domain that contains the receptor binding site and a membrane-proximal domain that shows a high degree of homology between strains and is referred to as the stem (Figure 1b).

Unfortunately, inactivated vaccines have only proven to be effective against homologous virus strains, owing to the apparent immunodominance of the highly variable HA head domain rather than the more conserved stem domain, with the majority of antibodies induced being directed towards this region [22,23,24,25,26]. Indeed, the HA head domain evolves at a faster rate than the stem domain [27], leading to a constant arms race to update and re-administer vaccines annually in order to keep up with this virus evolution. Additionally, such vaccines would likely offer little to no protection in the event of a zoonotic spillover event, meaning a new vaccine would have to be manufactured at a rapid speed. Ideally, a universal influenza vaccine capable of providing long-lasting protection against both seasonal infections as well as potential pandemic viruses should be available. If such an ambitious goal were to be achieved, new approaches to influenza virus vaccine development are required. This narrative review focuses on the next generation of influenza virus vaccines in the race towards universal protection.

## 2. Hemagglutinin Stem-Based Vaccines

Due to the immunodominance of the head domain, attempts to elicit a broadly protective immune response have focused on the more highly conserved HA stem domain [27]. Many monoclonal antibodies directed towards the HA stem domain have proven to be broadly protective, either within one of the phylogenetic groups outlined in Figure 1a or even providing universal protection from both group 1 and group 2 viruses [28,29,30,31,32,33,34,35]. This protection is mediated either through Fc effector functions or inhibition of the low pH-induced conformational changes necessary for membrane fusion.

To create these HA stem vaccine candidates, many approaches have been trialed. Initial studies designed HA stem constructs and delivered these as DNA or virus-like particle (VLP) vaccines [36]. Vaccination of mice with these VLPs was able to protect from homologous virus challenge and induced cross-reactive antibody responses capable of binding to heterologous HAs [36].

Subsequent studies utilized recombinant protein technology, incorporating trimerization domains (foldon or GCN4) as well as rational stabilizing mutations to produce soluble recombinant HA (rHA) stem vaccine candidates [36,37,38,39,40,41,42,43,44,45]. One such HA stem vaccine termed mini-HA #4900, which was based on H1 HA, was shown to provide protection against heterologous H1N1 and H5N1 challenge in a mouse model with no clinical symptoms or weight loss observed. Furthermore, sera from immunized mice contained antibodies able to bind to H1, H3, H5, H7 and H9 HAs, with neutralizing activity against an H5N1 virus also observed. These antibodies were also able to mediate antibody-dependent cellular cytotoxicity (ADCC), a key mechanism for broadly protective antibodies against influenza viruses. Upon challenge in cynomolgus monkeys, a significant reduction in fever was observed in vaccinated animals compared to control animals; however, no difference was observed in tracheal viral loads. This illustrates that well-designed HA stem constructs can induce broadly cross-reactive immune responses, though they do not necessarily reduce viral replication.

Stem-only HA constructs have also been designed from H3 HAs. Mallajosyula and colleagues [41] used sequence conservation to guide the design of multiple HA stem constructs and included the GCN4 isoleucine zipper or bacteriophage T4 foldon trimerization domain to enhance folding. Vaccination of mice with these constructs induced antibodies capable of binding to multiple H1, H3 and H7 HAs, and neutralizing a heterologous H3N2 pseudovirus. When assessed in an in vivo mouse model, however, only partial (40–50%) protection was observed following challenge with a homologous H3N2 virus, highlighting the challenges of achieving protection with stem-only HA constructs.

While most stem-only constructs provide protection from divergent strains, this was seen mostly within the same phylogenetic group. Inter-group protection has been achieved, however, with HA stem proteins based on H1 or H5 HAs [43]. In this study, vaccination with H1 or H5 vaccines provided protection from a H3N2 virus challenge in mice, with 40% and 80% survival after vaccination with H1 or H5 stem vaccines reported, respectively. While survival was evident after this H3N2 challenge, the viral load in the lungs was not reduced by vaccination, consistent with previous studies involving HA stem constructs.

To improve on the modest protection observed by many groups with headless rHAs, attempts to model pre-existing memory immune responses to the stem domain have been made. It was hypothesized that as some human populations have low levels of stem-reactive antibodies, immunization with stem-only rHA could selectively boost this antibody population, leading to broader protection [20,21,45]. To establish an animal model system which accounted for this memory, Wohlbold et al. immunized mice with a DNA vaccine encoding a chimeric HA consisting of the head domain from H9 HA and the stem domain from H1 HA. Such a vaccination strategy has been shown to induce a weak anti-H1 stem response [44]. When mice were primed with the DNA vaccine before immunization with stem-only rHA, complete protection was observed from homologous H1N1 challenge compared to just 40% from immunization with stem-only rHA alone. In a heterologous H6N1 virus challenge, 100% protection was seen when primed with the DNA vaccine, and 60% protection upon H5N1 virus challenge [44].

A similar approach was used by Ibanez et al. [46], where a stem-only HA vaccine based on an equine H3N8 influenza virus was utilized in DNA and subunit vaccine forms. Mice were vaccinated with a stem-only DNA vaccine (encoding the stem-only HA with a GCN4 trimerization motif) or a subunit protein vaccination boost (containing prokaryote-expressed HA stem with no trimerization domain), or prime-boost combinations of the two [46]. Using this strategy, both the DNA vaccine and the subunit vaccine, as well as DNA prime followed by the subunit boost, and vice versa, yielded 100% protection from homologous virus challenge. When a homosubtypic human H3N2 virus was used, only the regime using a subunit prime followed by a DNA vaccine boost showed 100% protection, with all other regimes showing only partial (20–80%) protection [46].

These studies suggest that a small amount of pre-existing anti-stem immunity, as is likely present in some of the human population, improves the effectiveness of stem-only influenza vaccines and provides a more cross-reactive antibody response. These results may provide a framework for the future application of headless rHAs as potential vaccine candidates in humans. Indeed, clinical trials assessing a stem-only vaccine candidate are currently underway (NCT03814720), which will be greatly beneficial to assess the impact of such vaccines in the human population.

## 3. Chimeric Hemagglutinin Vaccine Candidates

**Figure 2:**
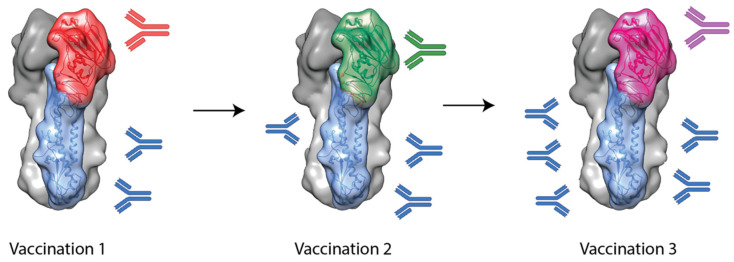
A schematic outlining chimeric HA-based vaccination approaches. A subject is immunized with a chimeric HA with an exotic head domain but a stem domain for the target virus. Following from this, a second vaccination can be administered with a chimeric HA with a consistent stem domain but another, different, exotic head domain. This can continue in order to selectively boost antibody responses towards the conserved stem domain while not boosting head-specific antibodies.

Another approach to inducing stem-specific antibodies involves the use of chimeric HAs (cHAs). These cHAs contain a stem domain from one subtype (e.g., H1 or H3) and a head domain from another, foreign subtype to which the subject is naïve (e.g., H5, H6 or H9) [47]. It was hypothesized that by sequential vaccination with cHAs with a common stem domain but different head domains, stem-specific antibodies would be selectively boosted and, thus, broader protection would result. This is outlined in Figure 2.

Studies using foldon-stabilized recombinant cHAs have shown that this technique has promise [48,49]. In these studies, mice were vaccinated three times with cHAs with common stem domains (e.g., H1 or H3 stem) but foreign head domains (e.g., H9, H6 or H5). Upon challenge with heterosubtypic viruses, from within the same HA phylogenetic group, cHA vaccination regimes were able to provide complete protection, though they failed to provide protection from intergroup challenge viruses [48,49]. This suggests the stem-specific antibodies induced by cHAs are mostly restricted to one hemagglutinin group.

While most efforts towards a universal influenza vaccine have focused on influenza A viruses, this cHA technology has been applied to influenza B viruses [50]. These candidate vaccines, which contained head domains from influenza A viruses with stem domains from influenza B viruses, could protect from a lethal challenge with diverse influenza B viruses from both Victoria and Yamagata lineages [50]. Analysis of the serum indicated that this protection was largely due to antibody effector functions such as ADCC rather than virus neutralization. This observation is consistent with data from studies on stem-specific monoclonal antibodies (mAbs), where their main in vivo mechanism of action was via antibody effector functions [28,51].

HA chimeras have been made as part of whole viruses as well as soluble recombinant HAs and, thus, can be used as split virus vaccines, live-attenuated influenza virus vaccines or subunit vaccines [47,48,52]. One such study utilized this cHA technology in a split virus vaccine modality [52]. Mice were first primed with a monovalent inactivated H1N1pdm09 vaccine before vaccination with H1 chimeric rHAs containing either a H5 or H8 head domain [52]. This approach induced higher stem-specific antibody levels when compared to current seasonal vaccines containing regular HA. These stem-specific antibodies were able to bind to H2 and H18 HAs in vitro [52]. The serum from immunized mice was able to provide complete protection from a heterologous virus challenge in a passive transfer experiment, where acceptor mice received sera from donor mice that were vaccinated with the chimeric regime outlined earlier before a challenge with a chimeric virus (containing a H1 HA chimera with an H9 head domain and an N3 NA protein—to ensure the only antibodies present from the donor mice were towards the conserved H1 stem domain) [52]. This study demonstrates that the cHA technology provides some level of stem-specific immunity that can protect from challenge with heterologous HA viruses.

Building on these pre-clinical data, data from a Phase I clinical trial were also recently published using this approach in the form of a prime/boost strategy with live attenuated and inactivated cHA virus vaccines [53]. Here, it was found that immunization with AS03 as an adjuvant elicited broadly cross-reactive antibodies directed towards the stem domain of HA. This important finding highlights the utility of the cHA approach in the human population.

With a similar objective as the cHA strategy, other approaches have focused on replacing more distinct antigenic sites in the HA head region rather than the entire domain [54,55]. By substituting immunodominant major antigenic sites of the H3 protein with corresponding sequences from exotic HAs, “mosaic” HAs are created. These HAs were then incorporated into reassortant viruses and subsequently inactivated for use as vaccines, where they induced broadly reactive stem-specific antibodies as well as head-specific neutralizing antibodies. Additionally, protection was afforded from challenge with historical H3N2 virus strains [54]. This approach has also been validated with influenza B viruses as a subunit vaccine approach, where cross-protection against heterologous influenza B virus strains was observed in mice [55].

## 4. Computationally-Optimized Hemagglutinin Vaccine Candidates

The use of computer software to create optimized consensus HA sequences for use in vaccines has shown some success. One such approach created computationally optimized broadly reactive antigenic (COBRA) HAs using in silico methods to find consensus H5 HA sequences that were predicted to capture common immune epitopes [56,57]. These HAs were then incorporated into VLP vaccines which were shown to induce a broader antibody response and reduce morbidity and viral titers compared to their non-optimized counterparts in mouse, ferret and non-human primate models [56,57]. Similar results have been seen for H1 HA COBRA VLPs [58], with broadly reactive antibodies induced upon vaccination of mice.

The COBRA HA technology has also been utilized in inactivated vaccine approaches for both H1 and H3 HAs [59]. While effective protection was observed, the breadth of the immune response to non-homologous viruses did not show much improvement over the non-optimized inactivated vaccine control groups.

“Mosaic HA” sequences have also been generated, again using in silico methods. For mosaic HAs, an algorithm was designed to select and optimize potential CD8 T cell epitopes [60]. The resulting sequence was then used as a vaccine delivered via a vaccinia vector system. This regime gave complete protection in mice from H5N1 virus challenge with multiple divergent strains as well as heterologous cross-protection to an H1N1 strain [60]. Protection against the heterosubtypic H3N2 challenge proved unsuccessful, however [60]. Interestingly, no detectable neutralizing antibodies were detected against the H1N1 strain used in the challenge, despite protection occurring. This suggests protection is mediated by either non-neutralizing broadly reactive HA antibodies or cellular immunity, which was seen to be induced by this vaccination approach [60]. Mosaic H5 HA vaccines have been tested in non-human primates also [61], though the technique has not been applied to other subtypes of HA.

## 5. DNA-Based Vaccines

To avoid many of the problems associated with egg-based inactivated influenza vaccines, nucleic acid-based approaches have been trialed. With this technology, an antigen-encoding gene in the form of a non-replicative expression plasmid is delivered into the host, where the host cells take up the nucleic and express the vaccine antigen. The host then presents the antigen to the immune system via major histocompatibility complex (MHC) pathways where a CD4 and CD8 T cell immune response against the endogenously expressed antigen can be mounted. This technology has the benefit of a long shelf life for vaccines, no requirement for growth of a live virus which reduces the infrastructure requirements and cost as well as the speed of nucleic acid technologies allowing rapid production of vaccine candidates. Additionally, altering the encoded gene is simple compared to other inactivated virus or recombinant protein-based technologies.

Initial trials with DNA vaccines used HA as an antigen, and reported DNA vaccines encoding H1 or H7 HA could provide protection from homologous virus challenge in mice and chickens, respectively [62,63]. The nucleoprotein (NP)-based DNA vaccine, however, could not provide protection even from homologous challenge [62]. Other studies report that NP-based DNA vaccination can provide partial protection from heterologous virus challenge [64]. Additional antigens such as the M2 protein have also been tested [65]. In this study, vaccination with the M2-based DNA vaccine induced M2-specific antibodies and protected against challenge with a lethal dose of homologous virus.

In order to improve on these results, other groups attempted multivalent approaches, with multiple antigens from a virus being delivered as a DNA vaccine. One such attempt fused the genes of HA and the matrix 2 protein ectodomain (M2e) from an H1N1 virus together and delivered this into mice as a vaccine. After vaccination, good serum antibody responses were observed against both HA and M2e, which translated to 100% survival from a heterologous H5N2 virus challenge [66]. Other approaches simply utilized multiple plasmids to encode for multiple antigens. Most studies utilized the NP, PB1 and M1 genes in this multivalent approach in an attempt to achieve cross-protection from diverse subtypes. Doing so has yielded reports of heterologous protection in mice, pigs, ferrets and non-human primates [67,68,69,70]. Even utilizing multiple strains of the same subtype of HA can result in broader immunity, albeit within the same subtype used in vaccination [71].

Much like recombinant protein approaches utilize stem-only HA vaccines to attempt to induce cross-protective immune responses, some groups have adopted this approach with DNA vaccine technology. Attempts have been made to deliver stem-only vaccine candidates in DNA vaccine forms [36]. Doing so induced a wider breadth of serum antibodies capable of binding to a broader range of HAs compared to using the full-length HA construct. Utilizing consensus HA sequences to create rHA-based vaccines showed some success, so the DNA vaccine-based approach with this technology was also trialed [72,73,74]. Various studies showed success for H1-, H5- and H7-based vaccines, as measured by an increase in the number of subtypes able to be bound by serum induced by vaccination with these consensus DNA-based vaccines. This correlated with an increase in neutralization [72,73,74].

After a wealth of pre-clinical studies, a phase I clinical trial was performed with a DNA vaccine candidate [75]. This vaccine candidate encoded HA from an H5N1 virus and was delivered as either a monovalent formulation (a plasmid encoding HA alone) or a trivalent formulation (a plasmid encoding HA, NP and M2). The monovalent formulation was able to elicit T cell responses against HA in 75–100% of the test subjects, and the trivalent formulation induced T cell and antibody responses to at least one antigen in 72% of individuals [75]. These data suggest that DNA-based influenza vaccines are at least partially effective at inducing an immune response in humans, though the effectiveness at preventing infection and disease has yet to be established.

Taken together, these data demonstrate the broad applicability of this DNA-based vaccine technology, especially when coupled with rHA-based findings. This suggests that DNA vaccines are a viable approach to a cheaper, easier way to manufacture influenza virus vaccines. The use of DNA vaccine technology allows this production of vaccine candidates quickly and easily, without the need to grow pathogenic viruses or optimize expression and purification steps, as would be the case for recombinant vaccine approaches. However, there are risks associated with DNA vaccines, including stable gene integration of transfected DNA into the host genome, which has oncogenic potential, as well as transfer of antibiotic-resistant genes present on the backbone of the plasmid to the host cell microbiome [76]. These safety concerns need to be considered as DNA vaccines make their way into human clinical use.

## 6. RNA-Based Vaccines

Other approaches to influenza vaccination have utilized mRNA-based rather than DNA-based vaccines. In such a system, the principle is very similar to that of DNA vaccines—an antigen-encoding mRNA molecule is delivered to a cell for subsequent expression of the vaccine antigen, whereby the host mounts an immune response against that expressed antigen. Such approaches have been shown to be successful for other viruses, with recent validation for SARS-CoV-2, where multiple mRNA vaccines have been progressed to licensure in under a year [77].

Early studies in the influenza vaccine field utilized liposomes to encapsulate mRNA encoding the NP from an influenza A virus. This vaccine was then administered to mice via a subcutaneous injection, where it was seen that virus-specific cytotoxic T lymphocytes (CTLs) were induced [78]. Other studies that used an HA mRNA-based vaccine encoding the full-length HA from the mouse-adapted PR8 H1N1 strain saw complete seroconversion as well as a CD4^+^ and CD8^+^ T cell response and protection from homologous challenge [79]. This mRNA-based vaccine approach was also applied to H3 and H5 viruses [79]. An NA-based mRNA vaccine also induced protection from homologous vaccination after a single dose [79]. To confer cross-protection, an NP-based mRNA vaccine was included. The use of NP mRNA resulted in 100% protection from homologous challenge and 80% protection from the closely related H5N1 virus. This cross-protection was shown to be mediated by T cells [79]. Additionally, the mRNA-based HA vaccines were immunogenic in both ferrets and pigs, with protection from homologous live virus challenge also established in pigs [79].

More recent studies by Moderna, developing a SARS-CoV-2 vaccine using this technology, have shown that using lipid nanoparticles (LNPs) to encapsulate their mRNA-based vaccines can increase the immune response for H7N9- and H10N8-based vaccines [80]. These LNP-encapsulated vaccines were able to provide protection from homologous virus challenge in mice and reduce viral loads in the lungs of live virus-challenged ferrets [80].

Another study utilizing full-length H1pdm09 HA in an LNP-based mRNA vaccine formulation elicited complete protection from homologous virus challenge as well as from heterologous PR8 H1N1 virus challenge [81]. Complete protection from a challenge with an H5N1-like virus (a 6:2 reassortant between PR8 and an H5N1 virus, with the HA and NA from the H5N1 virus and the internal genes from the PR8 virus) was also observed in vaccinated mice [81]. Analysis of the sera from these mice showed that appreciable stem-specific antibody levels were induced by this vaccine regime [81].

Further studies utilized a modified alphavirus self-amplifying RNA system, where the modified genome encodes for the RNA replication machinery (including the RNA-dependent RNA polymerase) of the alphavirus as well as genes for the antigen of interest [82]. The result is amplification of the RNA in the host cell and thus higher levels of antigen expression. This technique has been trialed in a mouse model with HA as the antigen of choice; however, vaccination with this candidate yielded only partial protection from disease upon homologous challenge [82]. Further studies utilizing this self-amplifying system demonstrated their ability for rapid production [83] and their dose-sparing advantages compared to synthetic mRNA alone [84].

Taken together, this evidence suggests that mRNA-based vaccines, which allow for rapid formulation and production, can induce protective antibody responses against homologous virus challenge. However, only limited cross-protection data exist, with partial H1-to-H5 cross-protection observed. Data analyzing the ability for this vaccine modality to induce protection from more divergent viruses would be ideal.

Given the speed of production for the mRNA-based SARS-CoV-2 vaccines, perhaps aiming for a universal mRNA vaccine for influenza is not required. Instead, in the event of an influenza pandemic, and with appropriate manufacturing infrastructure in place, an influenza mRNA-based vaccine could be updated to reflect the HA sequence of the novel pandemic virus. This could then be rapidly manufactured and deployed to impact the ongoing pandemic spread. This same capacity could be applied to seasonal vaccines to more rapidly manufacture new formulations with updated strains each year compared to traditional inactivated modalities. However, such an approach would likely require significant investment in infrastructure worldwide to ensure adequate manufacturing and distribution capabilities. The potential success of the SARS-CoV-2 mRNA vaccines could drive this change and see the use of mRNA vaccines becoming commonplace in the not-too-distant future.

## 7. Vector-Based Vaccines

Many studies have utilized viral vectors as vaccine delivery platforms for development of a universal influenza virus vaccine. In such vectored systems, a delivery virus is attenuated or rendered defective and a foreign expression cassette encoding the antigen of interest under the control of a mammalian promoter is inserted into the viral vector genome [85]. Upon vaccination, the virus vector infects the host cell, delivering the expression cassette for subsequent antigen expression and host antigen-specific immune response. Their use entails many advantages, including immunogenicity, induction of balanced antibody and T cell responses after vaccination, stability and ease of production [86,87].

Adenoviruses are a commonly used recombinant vector system (rAd) and are non-enveloped DNA viruses with a large (30–40 kilobase) double-stranded DNA genome [88]. To use adenoviruses as vectors, the E1A and E1B genes are deleted, rendering them defective, and replaced with the gene of interest [85]. Adenoviruses can infect a variety of cells, meaning that multiple rAd vaccine delivery routes are possible [89].

Many rAd-based vectors have been trialed as universal influenza virus vaccines. The first attempt utilized a replication-deficient rAd virus vaccine containing the HA gene from an H3N2 swine virus [90]. Vaccination of mice with this rAd-based vaccine induced high titers of neutralizing antibodies and showed 80% protection from challenge with a closely related H3N2 virus [90]. Interestingly, the serum could not neutralize the challenge virus in vitro, yet still provided partial protection. This was thought to be due to the induction of cell-mediated immunity [90]. Many other studies have utilized rAd-based vaccines expressing HA genes and shown protection from homologous viruses in both mice and ferrets as well as in vitro neutralization of a diverse range of heterologous viruses, albeit within the same subtype [91,92,93]. One study also demonstrated in vivo protection from a range of H5N1 virus strains in a mouse model [94]. Homologous protection, as well as partial intra-group heterologous protection, has been observed in pigs [95]. Interestingly, with rAd-based HA vaccination in the pig model, no vaccine-associated enhanced respiratory disease was observed upon the intra-group heterologous virus challenge, as is often the case with current industry-standard whole inactivated virus vaccines, suggesting a possible advantage of this vaccine modality [95].

To test the ability of rAd-based vaccines to induce more substantial cross-protection, mouse models involving challenge with heterologous H5N1 virus challenges after H1-based rAd NP-based vaccine were trialed [96]. In this study, partial protection was observed for one H5N1 strain, and 100% protection for another strain, indicating that this regime can induce cross-protection from distinct viral subtypes within the same phylogenetic group [96]. When this vaccine was tested in ferrets, however, this cross-protection was not reproduced [97].

In attempts to create a universal vaccine, many studies have been conducted with rAds expressing multiple viral antigens. In one such study, two rAds were created: the first expressing M1, NA and HA from the Spanish influenza H1N1 virus, and the second expressing M1, NA and HA from a highly pathogenic H5N1 virus isolate [98]. Vaccination of mice with a 1:1 mixture of both rAds was able to induce protection against H5N1 viruses from two distinct clades, suggesting a degree of intra-group cross-protection [98]. Other trials used chimeric gene products expressing M2e from H1N1, H5N1 and H7N9 viruses, combined with the HA from the same H1N1 virus [99]. Vaccination with this construct resulted in significant anti-M2e antibody titers as well as NP-specific CD8+ T cells [99]. Upon challenge with various H1N1 strains, both homologous and heterologous, 90% survival was seen [99]. Using ß2 microglobin knockout mice, which lack CD8+ T cells, and passive transfer experiments, it was shown that this protection was a result of both antibody and cellular immunity [99]. Combining a rAd-based vaccine expressing NP and M2 from a H1N1 virus with a DNA vaccine prime appears to result in increased cross-protection, as illustrated by the protection afforded against H1N1 and H5N1 viruses after vaccination of mice and ferrets [67]. Taken together, these data suggest that rAd-based vaccination based on multiple influenza viral antigens can be effective at providing protection from homologous and some heterologous virus challenges. However, protection against an inter-group challenge was not investigated.

The modified vaccinia virus Ankara (MVA) vector, an attenuated poxvirus, has also been used for development of a universal influenza virus vaccine. In this trial, MVA vectors expressing H5N1-derived NP, M1, M2, PB1 or a HA stem construct or a combination of HA stem, M2 and NP were developed [100]. Vaccination of mice with these constructs provided partial protection from H5N1, H9N2 and H7N1 challenge, with the NP-based MVA vaccine providing complete protection from H5N1 and H9N2 challenge and high-level partial (92%) protection from H7N1 challenge [100]. The HA stem/NP/M2 combination vector provided 100% protection from H9N2 and H7N1 challenge and 92% protection from H5N1 challenge [100]. These cross-protection results are promising, as they represent a wide range of group 1 and 2 influenza A viruses. This is perhaps the most promising viral vector-based vaccine approach, though further testing of this vaccine candidate in ferrets is required.

These trials all suggest that viral vector approaches for influenza virus vaccination are viable. In the event of a pandemic, this platform would allow for rapid production of a vaccine candidate based purely off sequence information, thus not requiring the growth of any live virus. Some more testing of this platform’s efficacy in ferrets or humans would be required to ensure that the cross-protective immune response seen in some mouse models is recapitulated in a more relevant model. This would have the added benefit of investigating whether any pre-existing immunity to the viral vector, or influenza viruses, as would be the case in most humans, could alter the efficacy of influenza-specific immune responses.

## 8. Peptide-Based Vaccines

Peptide-based vaccines have many advantages over other vaccine modalities, including their low manufacture cost and relatively uncomplicated production. However, peptides are relatively poor mimics of conformational antibody binding sites and so are mostly employed as the basis of vaccines aimed at eliciting cell-mediated immunity. Extensive research on peptide-based vaccines for influenza virus has been conducted, with most focus placed on T cell epitopes identified on conserved internal influenza virus proteins [101]. As such, some research has explored the potential for an influenza virus peptide-based vaccine aimed at stimulating a cellular immune response rather than a humoral response used by traditional vaccines.

One such peptide-based approach used computer software to predict CTL epitope peptides in proteins of the highly pathogenic H5N1 influenza A virus [102]. From these predictions, 35 peptides were purified and tested for in vivo cytotoxicity. This resulted in six peptides from PA, PB1, PB2 and M2, with potent CTL activation being taken forward into a mouse challenge model. This showed that intranasal vaccination with a combination of PA, PB1 and PB2 peptides could result in complete protection from a PR8 (H1N1) and H5N1 virus challenge [102].

A proprietary predictive algorithm was also used to predict human leukocyte antigen (HLA)-restricted epitopes to inform peptide-based vaccine design [103]. This study found peptides from M2 and NP proteins that were able to reduce weight loss but were not protective in a virus challenge model in transgenic humanized mice (which have human HLA-A2, the HLA that the peptides were designed to interact with) [103]. The peptides were, however, able to induce influenza-specific antibodies and peptide-specific T cells [103].

Another peptide vaccine strategy was based on the observation that the HA-specific mAb 12D1 neutralizes a broad range of H3 viruses and recognizes a linear peptide sequence in the HA2 subunit of HA [104,105]. A synthesized peptide spanning residues 76–130 of this long alpha-helix, encompassing the 12D1 binding site within residues 76–106, was still bound by 12D1 [104]. Following this result, it was hypothesized that immunization with a construct containing these residues would be able to induce a 12D1-like antibody response and, thus, a broad-spectrum protection from influenza viruses. To this end, residues 76–130 were conjugated to a Flag tag amino acid spacer followed by a cysteine residue for coupling to the keyhole limpet hemocyanin (KLH) carrier protein. Serum from mice immunized with this construct was able to bind to HAs from the H1, H2, H3, H5 and H7 HA subtypes, and protection from an H3N2 virus challenge was also observed [104]. Passive transfer experiments with sera from vaccinated mice suggested that this protection was at least partly antibody-mediated [104].

To date, most research has focused on developing a universal influenza virus vaccine strategy based on influenza A virus. Some research involving peptide vaccines against influenza B virus has shown promising results. One method involved conjugating a peptide containing solvent-exposed residues from the influenza B virus HA cleavage site (which is conserved between both Victoria and Yamagata lineages) to the outer membrane protein complex of *Neisseria meningitidis* as a carrier [106]. Upon vaccination of mice, this vaccine showed complete protection from lethal challenge with both Victoria and Yamagata lineage viruses. Additionally, this vaccine was able to reduce viral replication in the lungs compared to control groups [106].

Taken together, these data suggest that peptide-based influenza virus vaccines have potential to be broadly protective. The application of epitope-predicting algorithms to identify suitable peptide vaccine candidates shows promise; however, the use of adjuvants and carrier proteins is expected to be necessary to elicit a strong immune response. Further research into the formulations of these peptide-based vaccines could yield promising universal influenza virus vaccine candidates.

## 9. Non-HA-Based Vaccine Candidates

Although HA is the most attractive viral antigen for influenza vaccination, other antigens have been tested as vaccine candidates. One potential candidate is the M2 ectodomain (M2e). The M2 protein is a proton-selective ion channel expressed on the surface of the influenza virion and infected cells, and infection with influenza A virus generates a weak antibody response to the M2e protein [107,108,109]. The majority of the M2 protein is membrane embedded; however the N-terminus of the M2 protein complex exists as a highly-conserved ectodomain of 18–24 residues extending from the surface of the virus [110,111]. The conservation of the M2e sequence across influenza A virus subtypes makes it an attractive universal vaccine candidate. Indeed, clinical trials have been conducted to investigate the efficacy of an mAb that recognizes this exposed domain of M2e in human subjects infected with an H3N2 virus [112]. This study showed that the treatment was safe and well tolerated and produced a slight reduction in virus replication, demonstrating the effectiveness of anti-M2e antibodies and validating M2e as a target in an influenza vaccine candidate.

Early studies involving conjugation of M2e to the hepatitis B virus core protein (HBc) as a carrier in a subunit vaccine saw 90–100% protection from a lethal challenge with the H1N1 virus [110]. Clinical trials with this M2eHBc vaccine showed that it was well tolerated and elicited seroconversion [113]. Further studies have been conducted with various delivery methods such as VLPs, bacteriophage-based delivery, tandem repeats of M2e, recombinant proteins with tetramerization domains and gold nanoparticles [113,114,115,116,117]. In these studies, partial protection was observed at various levels from H1N1, H5N1 and H3N2 strains and some showed the ability to reduce virus replication in the lungs [114,115,117,118]. 

These studies clearly indicate that the M2e protein is a potential target for a broader influenza virus vaccine. However, this approach is likely only going to be successful for influenza A viruses, as the influenza B M2e is only 5–6 amino acids long, and thus, cross-reactivity with influenza A M2e antibodies is unlikely [119]. Additionally, the antigenicity of a subunit vaccine containing only this small protein is likely to be problematic. Perhaps formulations containing multiple antigens such as pre-fusion rHA and the M2e protein in combination would be a better option, as such formulations could provide sufficient protection from disease via anti-HA antibodies while also broadening subtype protection via anti-M2e antibodies.

To this end, recent studies have utilized protein nanoparticles (PNps) containing stem-only HA, stabilized by the GCN4 trimerization domain, and M2e peptides, stabilized by a GCN4 tetramerization domain [120]. The M2e portion of the nanoparticles is created first by ethanol desolvation, after which the soluble stem-only HA constructs are cross-linked using 3,3′disthiobis (sulfosuccinimidyl propionate) (DTSSP). This created an M2e nanoparticle core coated with stem-only HA [120]. Vaccination of mice with these PNps in either an H1 HA or H3 HA formulation, or a cocktail of both, showed robust seroconversion against M2e as well as the H1 or H3 components. The cocktail vaccine was also able to induce a cellular response as measured by interferon gamma-secreting splenocytes after exposure to antigen peptide pools. Subsequent virus challenge showed complete protection against virus strains with homologous HA components for both the H1 and H3 PNps. Furthermore, H1 PNps could fully protect against a reassortant H5N1 (rH5N1) virus challenge (H5 HA in a PR8 H1N1 virus backbone), and H3 PNps could fully protect against a reassortant H7N9 (rH7N9) challenge. When combined, the cocktail of H1 and H3 PNps together was able to provide full protection against H1N1, H3N2, rH5N1 and rH7N9 viruses in this model [120]. Interestingly, the M2e nanoparticle core alone was able to provide complete protection from H1N1 and H3N2 viruses and partial protection from rH5N1 and rH7N9 viruses [120].

Overall this approach appears promising and highlights the validity of using conserved regions in the M2e domain as an addition to existing vaccine technologies in order to provide broader protection. However, this approach is yet to move forward into clinical trials, and the lack of a head domain in the HA portion of these PNp vaccines presents the same issues as stem-only HA vaccine approaches, with a lack of neutralizing antibodies. In addition, manufacturing these PNp vaccines at scale will present challenges.

Another approach has combined a portion of the M2e protein with the NP protein. The NP of influenza A viruses is relatively conserved and is a target for CD8+ T cells during infection, so it presents an attractive target for vaccine development [121]. This approach utilized self-assembling NP nanoparticles delivered with a fusion protein of M2e and the papaya mosaic virus (PapMV) coat protein, which assembles around a single-stranded RNA (ssRNA) to form virus-like particles [122,123]. By combining the two antigens, protection against H1N1 and H3N2 virus challenges was observed in mice [123]. This approach further validates the notion that while antigens such as M2e and NP may not be capable of eliciting broad influenza virus protection alone, their combination with other antigens may prove effective.

**Figure 3:**
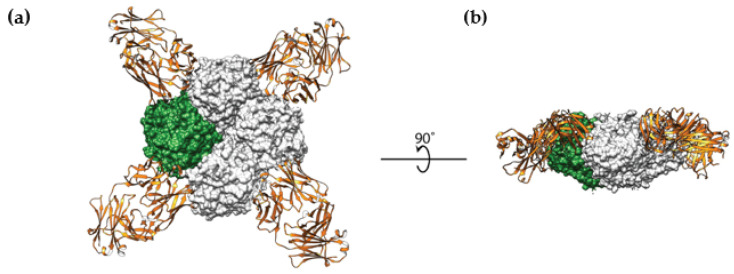
The structure of antibody CD6 in complex with influenza A virus neuraminidase (NA). (**a**) Top view of the NA–antibody complex. (**b**) Side view of the NA–antibody complex. Data retrieved from the Protein Data Bank (PDB ID 4QNP).

While NA has been a well-studied target for antivirals, vaccination strategies with NA have been somewhat neglected. Recent studies have uncovered a universal anti-NA antibody, capable of inhibiting all influenza A subtypes in vitro and providing complete heterosubtypic protection from H1N1 and H3N2 virus challenges in mice [17]. Numerous other antibodies have shown protection in animal studies [124,125,126,127]. One such antibody in complex with NA is illustrated in Figure 3. Additionally, current inactivated vaccines have been shown to induce an anti-NA antibody response in humans and ferrets, given that these formulations also contain NA antigen [128,129]. This NA content was also shown to be the dominant antigen in driving partial cross-protection from a lethal H5N1 virus challenge in the ferret model [130]. However, only the HA content of these vaccines was monitored and standardized, with NA content likely differing between preparations. NA should perhaps receive more consideration when it comes to both current seasonal vaccines as well as when designing a universal influenza virus vaccine in the future.

Recombinant ectodomain of NA has been used as a vaccine antigen, expressed in either insect cells via a baculovirus system or in yeast cells [131,132]. This protein was readily purified using affinity chromatography with an inhibitor of NA, oxamic acid. In both of these studies, vaccination with the recombinant NA was able to provide partial to complete protection from homologous challenge [131,132]. Further studies combined oxamic acid-purified NA, this time purified directly from detergent-disrupted virus, and used it to supplement traditional split, inactivated vaccines [133]. Doing so resulted in a balanced immune response towards HA and NA, equal to that of control groups receiving either HA or NA alone [133]. Upon challenge with homotypic virus (an H3N2 strain), no viral replication was observed and a greater reduction in viral replication was seen in groups challenged with heterosubtypic H3N2 [133].

More recent studies explored a similar concept, producing recombinant tetrameric NA using a GCN4-pLI tetramerization domain in place of the transmembrane domain [134]. Co-immunization of ferrets with this tetrameric NA with trimeric HA (achieved using a GCN4-pII trimerization domain) resulted in complete protection from homologous virus challenge [134]. The antibodies induced by NA vaccination were also able to inhibit NAs from other H1N1 viruses and, perhaps most importantly, an avian H5N1 virus [134]. This suggests that this vaccine approach could provide some form of cross-protection to other strains, though this was not tested in vivo. Interestingly, the authors observed that immunization with HA resulted in reduced viral replication, while immunization with NA alone resulted in a decrease in the clinical effects of infection, suggesting that HA- and NA-specific antibodies work in different ways. It was hypothesized that HA antibodies prevent infection completely, with NA antibodies allowing infection but blocking release of progeny virus and inducing other antibody-dependent immune effector functions. This phenomenon has been observed before, where an anti-NA-specific response was defined as “infection-permissive”, while still providing protection [135].

This evidence clearly points to NA as a target for influenza virus vaccination. It is also clear that current inactivated vaccines contain at least some NA as an antigen. There remain many questions regarding NA-based vaccination in influenza virus infection, however, concerning what epitopes are best to target on NA, the quality and quantity of NA in current vaccines, the optimal conformation of NA to induce a protective immune response and the breadth of protection by NA antibodies. In a similar fashion to the inactivation process destroying critical epitopes in HA in current inactivated vaccines, it is possible that critical NA epitopes are being destroyed too. Investigations utilizing recombinant NA vaccine antigens could help to answer some of these questions about the optimal conformation of NA and the antibodies it induces. Steps are being taken in the right direction to mitigate other issues such as the increased focus on NA as a vaccine target for influenza viruses, the development of assays to detect the quantity and quality of NA and more research into the breadth of NA immunity in the human population [136].

## 10. Conclusions and Outlook

Clearly, there is a need for improved influenza virus vaccines. The enormous pandemic potential of influenza viruses warrants serious concern, especially given the example of the damage that the SARS-CoV-2 pandemic has caused globally in 2020. Current vaccine modalities cannot be relied on to provide robust protection from seasonal strains, let alone any novel pandemic strains. The next generation of influenza vaccines would ideally be a universal solution—capable of providing complete protection from all influenza A and B viruses. While this is a lofty goal that is perhaps currently out of reach, research has been focused on vaccines that may lead us at least somewhat closer to this target. Such a vaccine would provide improved protection from drifted seasonal viruses that do not exactly match the vaccine strains, thereby limiting the impact of seasonal influenza virus infections while also providing some degree of cross-protection to novel, heterologous strains such as highly pathogenic avian influenza (HPAI) viruses that might cause a pandemic. This vaccine would likely need to target other viral antigens apart from HA. A novel vaccine containing multiple influenza virus antigens in combination, such as HA, NA and M2e, could be more likely to achieve the goal of improved protection from drifted and heterologous strains. This vaccine could also provide partial protection to slow the spread and impact of any potential pandemic strain that emerges, buying valuable time until a more potent strain-specific vaccine is made and is readily available. In such a case, rapid platform technologies capable of responding quickly upon virus discovery, such as mRNA vaccines, would prove valuable in the rapid production of a vaccine to slow the pandemic.

While the creation of a universal influenza virus vaccine is a technically challenging prospect, the task of proving efficacy in clinical trials also presents many logistical challenges. Due to the seasonal nature of influenza viruses, a universal influenza vaccine candidate would likely need to show multi-season efficacy, which would increase the cost and time of late-phase clinical trials. Additionally, the fact that the circulating virus strains change on an annual basis is also challenging. This would require any new vaccine candidate to either be effective regardless of the currently circulating strains or to be able to be manufactured quickly enough in order to have material ready for phase 3 clinical trials that matches the current season’s circulating strains.

A potentially more feasible approach involves addition of further components into existing vaccines to boost the breadth of protection without compromising protection against matched circulating strains. Peptide-based vaccines to boost reactivity to CTL epitopes and/or recombinant protein vaccines based on HA stem, M2e or NA may be compatible with existing inactivated virus or recombinant HA vaccines. Improvement on existing vaccines that already provide a level of protection against the matched influenza strains is a worthwhile objective and is likely to be more achievable relative to replacing existing vaccines. Ultimately, more research is required to investigate the novel technologies that are shaping the next generation of influenza virus vaccines, with the hope of one day finding a universal solution.
